# Therapeutic Effect of BDNF-Overexpressing Human Neural Stem Cells (F3.BDNF) in a Contusion Model of Spinal Cord Injury in Rats

**DOI:** 10.3390/ijms22136970

**Published:** 2021-06-28

**Authors:** Da-Jeong Chang, Hwi-Young Cho, Seyoung Hwang, Nayeon Lee, Chunggab Choi, Hyunseung Lee, Kwan Soo Hong, Seung-Hun Oh, Hyun Sook Kim, Dong Ah Shin, Young Wook Yoon, Jihwan Song

**Affiliations:** 1 Department of Biomedical Science, CHA Stem Cell Institute, CHA University, 335 Pangyo-ro, Bundang-gu, Seongnam-si 13488, Gyeonggi-do, Korea; dajoeng.c1024@gmail.com (D.-J.C.); hwangse3030@gmail.com (S.H.); db30922@hotmail.com (N.L.); moliver84@naver.com (C.C.); 2 Department of Physical Therapy, Gachon University, 191 Hambakmoero, Yeonsu-gu, Incheon 21936, Korea; hwiyoung@gachon.ac.kr; 3 Research Center for Bioconvergence Analysis, Korea Basic Science Institute, 162 Yeongudanji-ro, Ochang-eup, Cheongwon-gu, Cheongju-si 28119, Chungcheongbuk-do, Korea; hsys0307@kbsi.re.kr (H.L.); kshong@kbsi.re.kr (K.S.H.); 4 CHA Bundang Medical Center, Department of Neurology, CHA University, 59 Yatap-ro, Budang-gu, Seongnam-si 13496, Gyeonggi-do, Korea; ohsh72@chamc.co.kr (S.-H.O.); hskim626@cha.ac.kr (H.S.K.); 5 Department of Neurosurgery, Yonsei University College of Medicine, 50-1, Yonsei-Ro, Seodaemun-gu, Seoul 03722, Korea; 6 Department of Physiology, Korea University College of Medicine, Anam-dong 5-ga, Seongbuk-gu, Seoul 02841, Korea; 7 iPS Bio, Inc., 3F, 16 Daewangpangyo-ro 712 Beon-gil, Bundang-gu, Seongnam-si 13488, Gyeonggi-do, Korea

**Keywords:** spinal cord injury, contusion, neural stem cells, brain-derived neurotrophic factor (BDNF), functional recovery

## Abstract

The most common type of spinal cord injury is the contusion of the spinal cord, which causes progressive secondary tissue degeneration. In this study, we applied genetically modified human neural stem cells overexpressing BDNF (brain-derived neurotrophic factor) (F3.BDNF) to determine whether they can promote functional recovery in the spinal cord injury (SCI) model in rats. We transplanted F3.BDNF cells via intrathecal catheter delivery after a contusion of the thoracic spinal cord and found that they were migrated toward the injured spinal cord area by MR imaging. Transplanted F3.BDNF cells expressed neural lineage markers, such as NeuN, MBP, and GFAP and were functionally connected to the host neurons. The F3.BDNF-transplanted rats exhibited significantly improved locomotor functions compared with the sham group. This functional recovery was accompanied by an increased volume of spared myelination and decreased area of cystic cavity in the F3.BDNF group. We also observed that the F3.BDNF-transplanted rats showed reduced numbers of Iba1- and iNOS-positive inflammatory cells as well as GFAP-positive astrocytes. These results strongly suggest the transplantation of F3.BDNF cells can modulate inflammatory cells and glia activation and also improve the hyperalgesia following SCI.

## 1. Introduction

Spinal cord injury (SCI) has very limited therapeutic options currently, despite its devastating pathology. In SCI, the initial direct damage occurs on the cord, followed by a secondary cascade of breakdown of blood–spinal cord barrier (BSCB), inflammation, and excitotoxic damage that considerably worsens the extent of cell loss, particularly in neurons, oligodendrocytes, astrocytes [1,2,3]. In the incomplete SCI, the loss of oligodendrocytes is prevalent, causing sparing axons that are unable to conduct normal neurotransmission [4]. Neural stem cells (NSCs) have the capacity to proliferate and have multipotent potential to differentiate into the major cell types in the central nervous system (CNS), including neurons, astrocytes, and oligodendrocytes. Transplantation of NSCs can replace the lost neural cells in the environment of injured spinal cord [5]. Therefore, several studies have reported functional recovery by transplantation of various neural stem/progenitor cells in animal models of SCI [6,7,8]. Transplantation of NSCs inhibits the activation and proliferation of T cells. They attenuated inflammation and reduced demyelination and axonal pathology, thereby improving the clinical severity in acute and chronic encephalomyelitis (EAE) [9]. Transplantation of NSCs can also reduce inflammatory and gliosis reactions that contribute to demyelination, failure of axons, and cavitation following spinal cord injury [10,11,12]. 

Recently, intrathecal transplantation of cells has been regarded as an alternative way that would cause minimal damage to the injured area of the spinal cord [13,14] by reducing the risk of additional neurologic damages caused by direct local injection [15]. The therapeutic benefits of immortalized NSC cells are thought to be primarily related to their secretion of soluble factors that can provide neural protection and support in neurological disorders [16,17]. The objective of this research was to determine whether the intrathecally transplanted F3.BDNF cells can survive and migrate to the contusion-injured spinal cord and then improve functional deficits of impaired hindlimbs. 

## 2. Results

### 2.1. Tracking of F3.BDNF Cells in a Rat Model of Contusive Spinal Cord Injury

**Figure 1:**
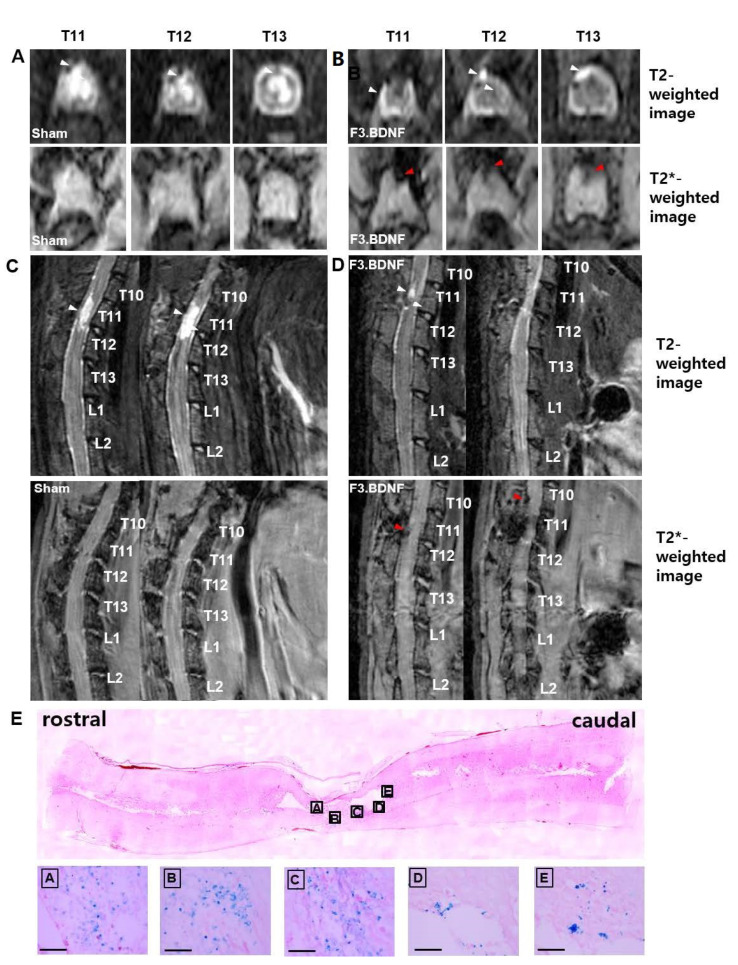
Detection of Feridex^®^-labeled F3.BDNF cells on MR images. (**A**,**C**) Sham group, (**B**,**D**) F3.BDNF group (*n* = 3 each). T2-weighted MR image on the top line in (**A**,**B**) and T2 *-weighted MR image on the bottom line in (**A**,**B**) at 72 days after spinal cord injury. The red arrowhead indicates the migrating Feridex^®^-labeled F3.BDNF cells toward the contusive injured spinal cord at the T11, T12, and T13 of axial levels and T10 and T11 of sagittal T2 *-weighted MR images. The white arrowhead indicates the contused spinal cord area. The hyperintense lesions in T2-weighted MR images at 72 days after spinal cord injury showed a significant difference between F3.BDNF group and sham group. (**E**) Histological distribution of labeled F3.BDNF after transplantation. Prussian blue staining shows that labeled F3.BDNF cells were migrated away from the injection site and dispersed throughout the injured spinal cord. Scale bar: 100 μm.

Contusive SCI animal models were induced at the T11 level in SD rats, and 1 × 10^6^ F3.BDNF cells or culture media were injected into the T9 at 7 days after injury. The F3.BDNF cells were labeled with ferumoxides (Feridex^®^, Taejoon Pharm, Seoul, Korea)—protamine sulfate complexes for 16h in vitro. Following incubation in the Feridex^®^-protamine sulfate complexes labeled F3.BDNF cells were analyzed to confirm the uptake of Feridex^®^ particles using Prussian blue staining (Figure S1). In the medium-treated SCI rats (sham group), significantly swelled spinal cord was found on T2–weighted imaging at 2 days after SCI. The spinal cord remained in a swollen state until 58 days after injury (Figure 1A,C). The swelling of the spinal cord increased in size at 58 days compared with 2 days after injury. The migration of labeled F3.BDNF cells toward the epicenter were monitored on MR images. No Feridex^®^-labeled cells were detected on T2 *–weighted imaging at 2 days after transplantation, but positive signals were detected at 58 days after transplantation (Figure 1B,D). By contrast, no positive signal was detected in the sham group (data not shown). Following MR imaging of spinal cords, imaged tissues were confirmed by Prussian blue staining to detect the transplanted F3.BDNF cells within the area of MRI-positive signal condensation. On sagittal sections of the lesion, Prussian blue-positive cells were found in both white and gray matters of the injured spinal cord region. Transplanted F3.BDNF cells were also found in the transplantation site (T9) as well as in the injury site (T11), in accordance with the MR images (Figure 1E). 

### 2.2. Survival and Differentiation of Transplanted F3.BDNF Cells in the Injured SCI Rats

**Figure 2:**
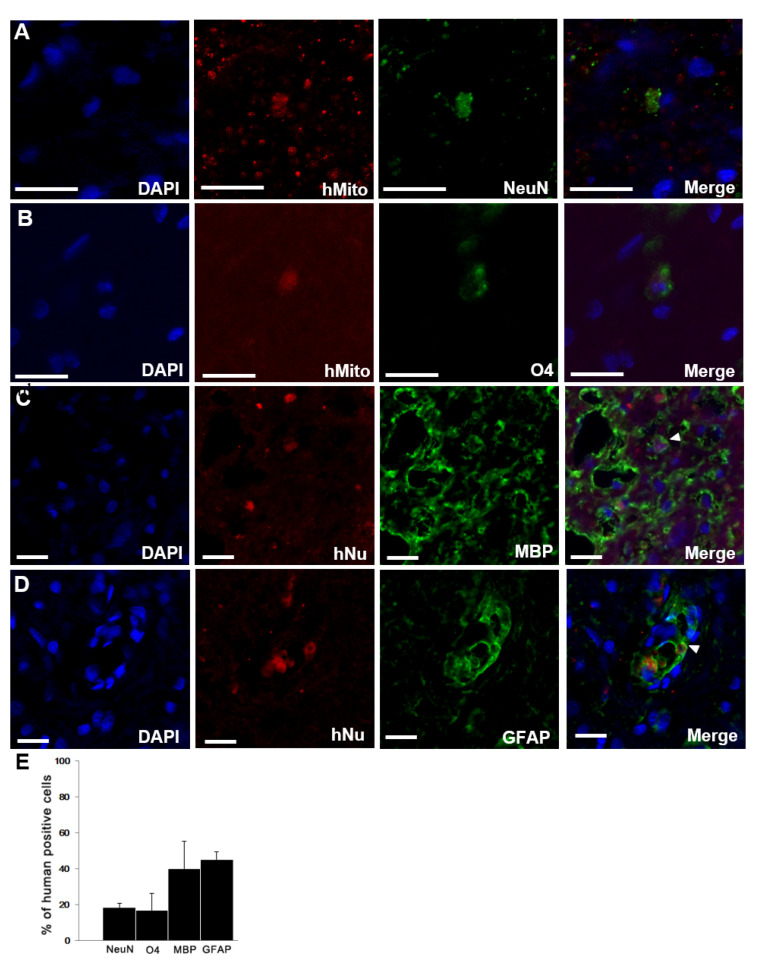
Differentiation of transplanted F3.BDNF cells in SCI rats. Transplanted F3.BDNF cells, co-labeled with either anti-human-specific mitochondria (hMito) or nuclei (hNu), were differentiated into (**A**) NeuN, (**B**) O4, (**C**) MBP, and (**D**) GFAP in vivo. Scale bar = 20 μm (**E**) quantitative analysis of the differentiated phenotypes into NeuN-positive neurons, O4-positive immature oligodendrocytes, MBP-positive oligodendrocytes, and GFAP-positive astrocytes. Data are shown as means ± S.E.M (*n* = 3 each).

We found that the transplanted F3.BDNF cells were differentiated into several neural lineage cell types around the lesion site in the injured spinal cord by using immunohistochemistry. Transplanted F3.BDNF cells were extended into both gray and white matter. They were identified as human-specific hMito- or hNu-positive cells. Among the differentiated cell types, NeuN-positive neurons were 18.4 ± 2.03% (Figure 2A), O4-positive oligodendrocytes were 16.67 ± 9.62% (Figure 2B), MBP-positive oligodendrocytes were 39.94 ± 15.34% (Figure 2C), and GFAP-positive astrocytes were 45.05 ± 4.13% (Figure 2D). These results indicate that transplanted F3.BDNF cells were differentiated into neurons, oligodendrocytes, and astrocytes in the injured spinal cord (Figure 2D).

### 2.3. Connection of the Transplanted F3.BDNF Cells in the Injured Spinal Cord to the Host Neuronal Networks

**Figure 3:**
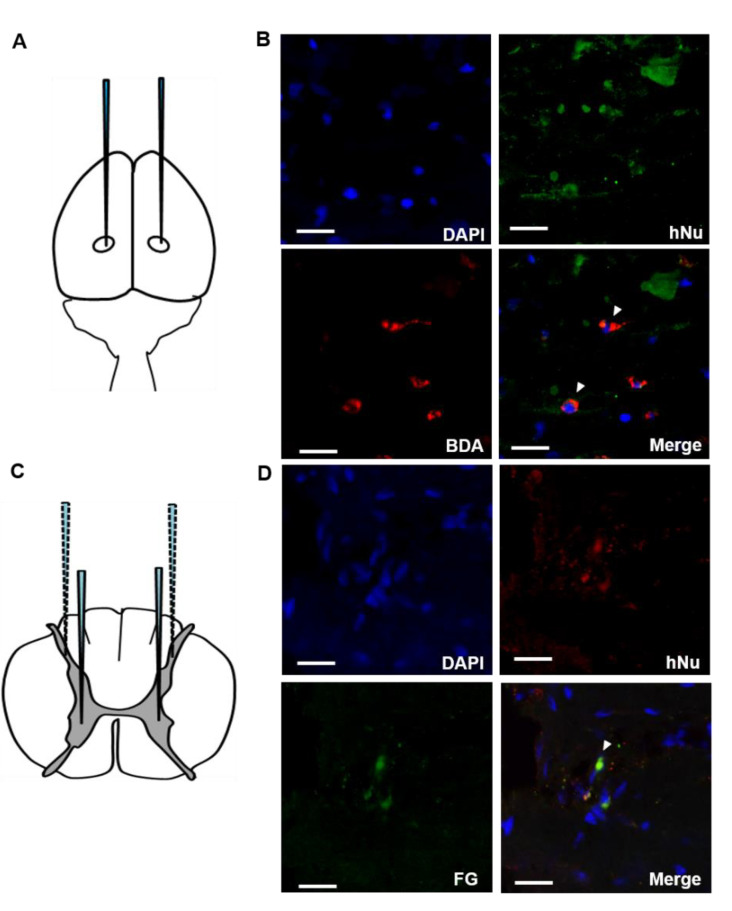
Tracing of transplanted F3.BDNF cells in vivo. (**A**,**C**) A schematic illustration showing bilateral injection of BDA in the motor cortex and bilateral injection of FG in the ventral horn and dorsal horn. (**B**) Two weeks later, BDA (red) was found in the F3.BDNF cells (green) that were identified with anti-hNu antibody. (**D**) One week later, F3.BDNF cells were co-localized with FG (green). Scale bars = 20 μm. Abbreviations: BDA, biotinylated dextran amines; FG, fluoro-gold; hNu, human nuclei antigen; DAPI, 4′, 6-diamidino-2-phenylindole.

To determine whether the transplanted F3.BDNF cells can connect to the host neural cells, we used anterograde and retrograde tracers in two independent experiments. First, we injected the anterograde tracer, BDA [17,18], into the right and left sensorimotor cortex at three points of each hemisphere 10 weeks after SCI and sacrificed the animals at 12 weeks. BDA-labeled corticospinal tract (CST) fibers crossing over the midline were found on the opposite side of the spinal cord [19]. The descending CST controls the movements of limbs [20]. Transplanted F3.BDNF cells were detected with hNu in the rostral part but not in the caudal part of the injured spinal cord, which was labeled by a BDA tracer (Figure 3A,B). Second, we also injected the retrograde tracer, FG [17], into the intact caudal region, skirting around the spinal cord injury lesion. The hNu-positive cells were found mainly around the injury one week after FG injection, and they were labeled with FG and were extended toward the rostral direction on horizontal sections (Figure 3C,D). The FG tracer was transported from the intact host neurons and was taken up to F3.BDNF cells. Taken together, these results strongly suggest that transplanted F3.BDNF cells were connected to the host neural networks in the contusive spinal cord injury by using anterograde and retrograde tracers.

### 2.4. Attenuation of Demyelination and the Areas of Cystic Cavity in the Injured Spinal Cord by Transplanted F3.BDNF Cells

**Figure 4:**
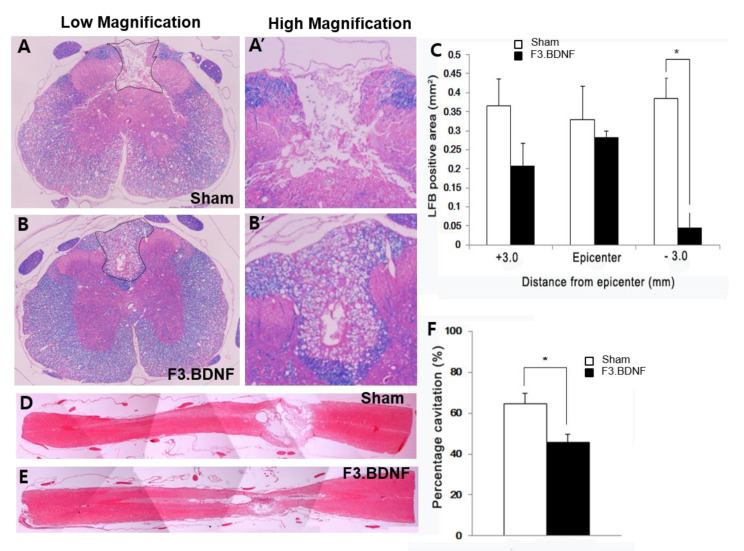
Effects of transplanted F3.BDNF cells within the injured spinal cord. (**A**,**B**) Representative LFB staining images of a transverse sectioned spinal cord at the lesion epicenter at 10 weeks after transplantation. (**A’**,**B’**) Higher magnification images of the boxed areas in A and B. The nuclear staining was followed by counterstaining with eosin. (**C**) For quantitative analysis of the spinal cord area, LFB-stained axial sections at 0.3 mm caudal and 0.3 mm rostral to the lesion were measured. (**D**,**E**) Horizontal sections of the spinal cord at the lesion epicenter were significantly reduced in the F3.BDNF groups than the sham group. (**F**) Quantitative analysis of the spinal cord injured area measured in H&E stained horizontal sections through rostral and caudal from lesion epicenter (*n* = 3 rats per group, *: *p* < 0.05) Abbreviations: H&E, hematoxylin-eosin; LFB, luxol fast blue staining.

To observe the beneficial effects of F3.BDNF cell transplantation on spinal cord repair, the severity of injury at the epicenter site was evaluated histologically at 10 weeks after SCI. Luxol fast blue (LFB) staining revealed a significant decrease of the demyelinated dorsal column in the F3.BDNF group, compared with the sham group (Figure 4A,B). We also examined the atrophic changes of the injured spinal cord by eosin staining. Quantitative analysis revealed significant differences in the caudal area but not in the rostral and epicenter of the impaired spinal cord between the F3.BDNF group and the sham group, suggesting that the F3.BDNF cell transplantation prevented atrophy of the injured spinal cord (*P* < 0.05; Figure 4C). We measured the area of cystic cavity using hematoxylin–eosin (H&E) staining (Figure 4D,E). In the F3.BDNF group, the average area of cystic cavity was significantly smaller than that in the sham group (*P* < 0.05; Figure 2F), indicating that survival of transplanted cells could prevent secondary degeneration of the spinal cord tissue after SCI and reduce the volume of impaired spinal cord tissue by providing trophic effects.

### 2.5. Reduction of the Host Inflammatory Responses by Transplanted F3.BDNF Cells

**Figure 5:**
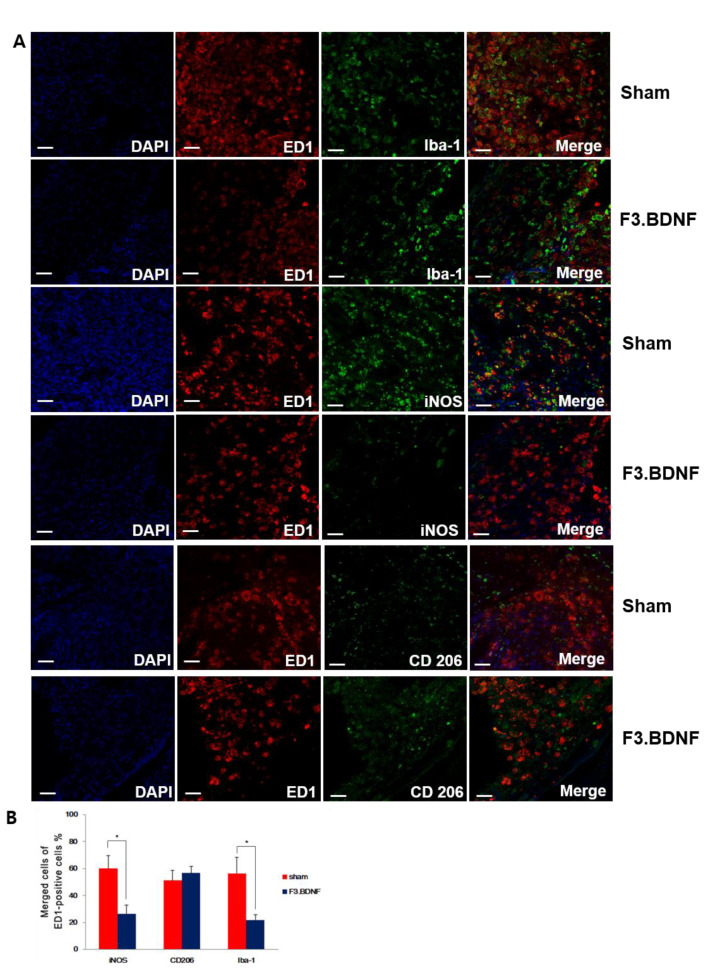
Histological analysis of inflammation. (**A**) Immunofluorescence staining showing differences in the expression of microglia marker Iba-1, in which M1 phenotype for classically activated macrophages (iNOS) and M2 phenotype for alternatively-activated macrophages (CD 206) were co-localized with ED1 (red) at 10 weeks after F3.BDNF transplantation in the injured spinal cord. In the F3.BDNF group, the number of iNOS-positive cells was decreased, and the number of CD206-positive cells was increased compared to those of the sham group. The nuclei were counterstained with DAPI (blue). (**B**) Percentage of merged cells of ED1 -positive cells around the epicenter region (scale bars = 20 μm; * *p* < 0.05).

We next investigated the effects of transplanted F3.BDNF cells on the expression of inflammatory cells 10 weeks after SCI. High levels of expression in Iba-1 for the activated microglia, iNOS for classically activated macrophage (M1 phenotype), and CD206 for alternatively activated macrophages (M2 phenotype) with ED1 for microglia/macrophages were observed at the injury site (Figure 5A). Contusion injury to the spinal cord leads to chronic activated microglia/macrophages. The number of Iba-1 positive cells with co-expressing ED1 was found mainly around the injury site and showed a significantly decreased expression level between the transplanted F3.BDNF and the sham group (21.83 ± 3.78% vs. 56.24 ± 12.21%, *p* < 0.05). The significant difference (*p* < 0.05) in the proportions of iNOS co-expressing ED1-positive cells between the transplanted F3.BDNF (26.34 ± 6.54%) and the sham group (60.01 ± 9.57%) showed that F3.BDNF cell transplantation apparently leads to the decrease of the classically activated macrophages (M1 phenotype). However, we did not observe a statistically significant difference in the relative proportion of CD206-positive cells (M2-like) co-expressing ED1-positive cells between the F3.BDNF and the sham groups (56.66 ± 4.87% vs. 51.15 ± 7.22% of the ED1-positive cells, respectively; Figure 5B). Transplantation of F3.BDNF cells were associated with a significant reduction in astrogliosis within the lesion margins. Quantitative analysis on the GFAP-positive astrocytes around the margins of the injured spinal cord revealed a considerable difference between the transplanted F3.BDNF group and the sham group injury sites (18.46 ± 1.94% vs. 81.55 ± 3.38%, *p* < 0.001, Figure S2). Taken together, these findings strongly suggest that transplantation of F3.BDNF cells can significantly reduce the inflammatory response and glial scar formation following spinal cord injury.

### 2.6. Improvement of Hindlimb Motor Function and the Reduction of Mechanical Hyperalgesis by the Transplanted F3.BDNF Cells

**Figure 6:**
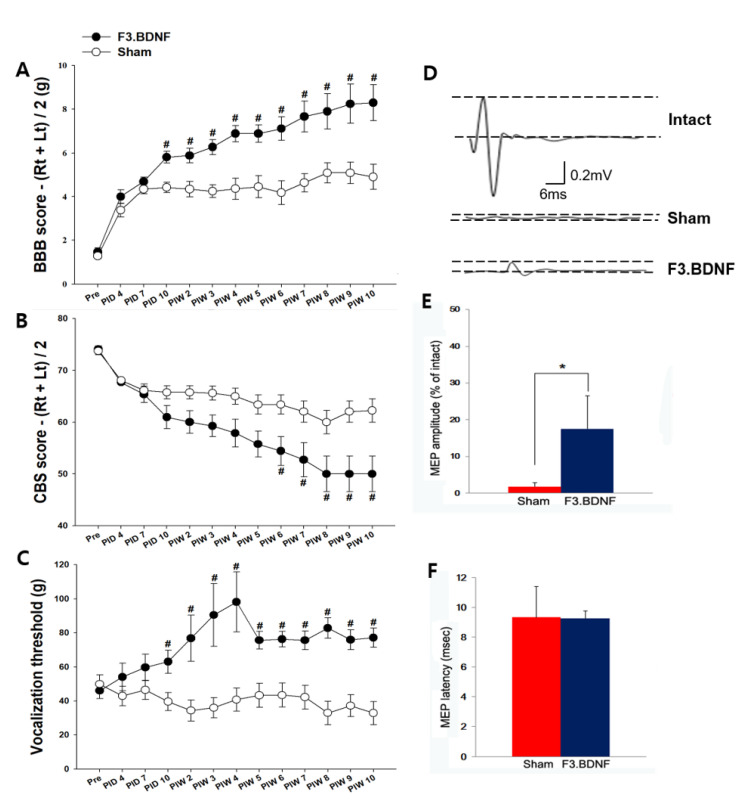
Transplanted F3.BDNF promoted motor functional and electrophysiological recovery after SCI. (**A**) Motor function in the hindlimbs was assessed by the BBB scores up to 10 weeks post-SCI. (**B**) Neurological assessment of functional deficit was measured by CBS test up to 10 weeks post-SCI. (**C**) Pain-related behavior was measured in vocalization threshold using Von Frey filaments in response to a rat mechanical stimulus applied to the back skin. The functional behavioral tests in the F3.BDNF group (*n* = 13) were significantly higher than that in sham group (*n* = 13). Data are presented as means ± SEM. Behavioral tests were analyzed by three observers who performed the blind tests independently. (**D**) Electrophysiological analysis was performed 80 days after SCI. The right motor cortices were stimulated, and MEP amplitudes were recorded from the left hamstring muscles. The data of the MEP indicate the latency for the time of stimuli from the onset to peak and amplitude. MEP waves were detected in most of the F3.BDNF group, whereas they were not detected in the sham group. (**E**) Relative values of the mean MEP amplitudes. Values are expressed as percentages of those in the intact animals (*n* = 3). Mean relative MEP amplitudes in the F3.BDNF group (*n* = 4) was significantly higher than that in the sham group (*n* = 3). (**F**) Relative values of the mean MEP latency. Mean relative MEP latency in the F3.BDNF group was not significantly different. *, *p* < 0.05; Data are means ±SD. Abbreviations: PID, post injury day; PIW, post-injury week. # *p* < 0.05.

We next monitored the changes of impaired hind limb motor function using BBB [21,22] and CBS [23] tests for 10 weeks after SCI. Previous studies have already shown that transplantation of F3.BDNF cells can be beneficial for behavioral recovery in ICH (intracerebral hemorrhage) and MCAo (middle cerebral artery occlusion) models [16,24]. All our SCI animals showed a complete hindlimb paralysis on the same day after the induction of SCI. From 3 days after transplantation, the transplanted group showed a slight movement from the joints of hindlimbs (i.e., hip, knee, and ankle) (n = 13) compared to the sham group (n = 8). Transplanted animals showed hindlimb locomotor recovery gradually from 2 weeks post-injury (*p* < 0.05) (Figure 6A). They showed a significant difference in BBB and functional recovery compared to the sham group at 10 weeks post-injury. Transplanted animals showed significantly lower CBS scores (*p* < 0.05) than the sham group from 6 weeks post-injury (Figure 6B). Transplanted animals were able to spread toes almost perfectly when the observer lifted up the animals. They showed an improved withdrawal reflex and placing reflex compared to the sham group from 6 weeks post-injury. We next investigated whether transplantation of F3.BDNF cells can play a role in neuropathic pain. When SCI animals were stimulated by the von Frye filament, vocalization response of individual variations can be created as to the specific dermatome [25]. The extent of the vocalization threshold in response to stimuli of the von Frye filament can be recorded. Interestingly, the F3.BDNF group showed a significant reduction in hyperalgesia, which was induced by SCI, compared to the sham group (*p* < 0.05) (Figure 6C). To address the effect of stem cell transplantation on the expression of GABA in neuropathic pain, we analyzed the difference in GABA expression between the sham group and F3.BDNF group in the dorsal horn, Although GABA immunoreactivity was decreased in the injured dorsal horn, no significant difference of GABA expression was observed in the injured dorsal horn following transplantation of F3.BDNF cells (Figure S4). Next, to evaluate the recovery of descending pathways from the motor cortex to the hindlimb motor neurons, we monitored motor-evoked potential (MEP) at 12 weeks after SCI. To conduct this, we stimulated motor cortices electrically and recorded MEP amplitudes from the hamstring muscles. The MEP amplitudes in the F3.BDNF cell-transplanted group were significantly higher than those in the sham group (Figure 6D,E). However, the latency of MEP response in the F3.BDNF cell-transplanted group was not significantly different compared to the sham group (Figure 6F). These results suggest that transplantation of F3.BDNF cells into the injured spinal cord stimulates the functional recovery of hindlimbs.

## 3. Discussion

This study demonstrates that transplantation of BDNF-overexpressing HB1.F3 cells (F3.BDNF) into a contusive SCI animal model promotes functional recovery in the hindlimb movement. Transplantation of neural stem cells in the SCI model has been reported to improve functional recovery [7,26]. Transplantation of neural stem cells can also lead to functional improvement in diverse neurological disorders, including ischemic brain injury [27,28], Huntington’s disease [29], and Parkinson’s disease [30,31]. However, the precise mechanisms for these apparently diverse roles have yet to be elucidated. F3.BDNF cells have been reported that they can lead to functional recovery and neuroprotection in an animal model of intracerebral hemorrhage [16]. In this study, we transplanted F3.BDNF cells via intrathecal delivery method into the T9 level of SCI model. The intrathecal delivery route for cell transplantation is a minimally invasive technique. Transplanted F3.BDNF cells into T9 survived and were migrated toward the lesion site on MR image (Figure 1). According to previous reports, transplanted F3 NSCs exhibited a trophism by attractant signals, including SDF-1, VEGF, or SCF emitted from the injury sites in the CNS [32,33,34]. Migrating F3.BDNF cells could simultaneously express human BDNF and differentiate into neurons and glia lineages in the spinal cord following contusive injury (Figure 2). It is known that BDNF can contribute to ensuring the long-term survival of newly generated neurons [35]. The migrated cells were located in the gray and whiter matters of the spinal cord, as well as in the contusion-injured sites. Indeed, transplanted F3.BDNF cells showed a connection to the host neurons located in the spinal cord, as shown by tracing of BDA or FG (Figure 3). Some studies reported that human iPSC-derived NPCs and hiPS-It-NES (long-term self-renewing neuroepithelial-like stem) graft exhibited graft-host interconnection properties with host descending tracts, CST, using BDA [36,37]. These results suggest that F3.BDNF cells play a crucial role in functional improvement by differentiating into functional neurons in the spinal cord and survive for an extended period of time in vivo. BDNF can also support axonal growth and regeneration after spinal cord injury in adult rats [37,38]. In this study, transplantation of F3.BDNF cells led to a significant locomotor recovery from 3 days to 10 weeks in the BBB test after SCI (Figure 6A). In the CBS test, the F3.BDNF group showed a significant improvement of functional neurologic impairment from 6 weeks after SCI (Figure 6B). Recent studies showed that neurons derived from transplanted human NSCs or human iPSCs can integrate into the injured spinal cord and form synaptic connections with host neurons in the injured spinal cord [6,39,40,41]. In the present study, transplantation of F3.BDNF cells promoted the healing of the severely injured spinal cord tissue, including the overall extent of injury, demyelination and the change of inflammatory response, by preventing the secondary degeneration. In the BDNF-hypersecreting hMSC (mesenchymal stem cells) group, the increased CST fiber sprouting in the rostral dorsal funiculus tissue regions suggests that BDNF induces increased plasticity within the spinal cord [34]. On the other hand, the engrafted NSCs expressing BDNF simultaneously improved behavior functions significantly in adult rats with spinal cord transection (SCT). However, no effect on CST regeneration in the injury site was observed [42]. Chronic neuropathic pain following a spinal cord injury is common and very important to treat, but it is a lack of understanding of the mechanisms. Recent studies consistently report that activation of astrocytes and microglia contributes significantly to central neuropathic pain following SCI [43,44]. The F3.BDNF cells reduced hyperalgesia in response to mechanical stimulations in the SCI model (Figure 6A). This effect may be due to the reduction of astrocyte reactivity and the change of local immune cells in animals transplanted with F3.BDNF cells. The glial cells produce numerous pro-inflammatory cytokines such as interleukin 1β (IL-1β), IL-6, and tumor necrosis factor α (TNF-α). These cytokines stimulate chronic pain in the spinal cord [45]. By contrast, the glia scar inhibitor induces efficient axonal regeneration and functional compensation after traumatic CNS injury [46,47]. The role of neuroinflammation after SCI is controversial, given the positive and negative aspects of macrophage/microglia and cytokine [48]. However, numerous studies suggest that depletion or neutralization of neutrophils and macrophages promotes neuroprotection and improves functional recovery [46,49,50]. The activated microglia and macrophages can cause initiation of axonal injury, secondary tissue damage, and progressive cavitation through the release of cytokines, free radicals, eicosanoids, and proteases following SCI [51,52,53]. Another possible mechanism is that a hypofunction of GABAergic tone in the spinal dorsal horn can lead to central neuropathic pain after SCI [54,55]. In our previous study, we reported that F3.BDNF cells can be differentiated into GABAergic neurons in the ischemic animal brain [19], and a similar result was observed in the injured spinal cord (Figure S3). We then analyzed the difference in GABA expression between the sham group and F3.BDNF group in the dorsal horn and found that GABA immunoreactivity was decreased in the injured dorsal horn. However, no significant difference in GABA expression in the injured dorsal horn following transplantation of F3.BDNF cells were observed (Figure S4). Therefore, there is a possibility that neuropathic pain was alleviated in SCI models is likely due to the fact that transplanted F3.BDNF cells were differentiated into GABAergic cells partially. In electrophysiological experiments, the transplanted rats showed a significant recovery of MEP amplitude after SCI (Figure 6D–F). In the sham group, we did observe a slight MEP amplitude/latency due to the incomplete failure of axonal regeneration in the impaired spinal cord. The recovery of MEP amplitude can be attributed to the reconnection of the axons, leading to the recovery of motor function following F3.BDNF cell transplantation. 

The present results demonstrate that transplantation of F3.BDNF cells intrathecally is a useful method for efficient cell delivery and therapy in a rodent model of SCI, causing the minimally traumatic method of transplantation when F3.BDNF cells are implanted into the cerebrospinal fluid. Labeled cells with Feridex^®^ can be monitored using MRI, which allows real-time monitoring of stem cell migration in vivo. Transplanted F3.BDNF cells survived and were differentiated in the injured SCI model and improved hindlimb movement. Taken together, these results strongly suggest that transplanted F3.BDNF cells can provide therapeutic benefits following SCI. Although a similar study was reported using PSA-NCAM sorted neural progenitors from BDNF-overexpressing mouse embryonic stem cells in a mouse model of SCI [56], our study using human neural stem cells overexpressing BDNF in a rodent model of SCI with intrathecal delivery, visualized by MRI, will provide a useful experimental strategy for human applications in the future.

## 4. Materials and Methods

### 4.1. Cell Culture

The HB1.F3 cells (F3) used in this study were originally prepared from telencephalon tissue in a 15-week gestational human fetal brain, followed by immortalization using the epitropic retroviral vector encoding *v-myc,* as described previously [57]. HB1.F3 cells (F3) expressed phenotype-specific markers for NSCs and neural progenitor cells, and cell type-specific markers for neurons and astrocytes [57]. F3.BDNF cells were established by a pBABE-BDNF vector as previously reported [16]. F3.BDNF hNSCs were grown in 0.2% gelatin-coated tissue culture dishes (BD Falcon^TM^, Corning, New York, NY, USA) and maintained in Dulbecco’s modified Eagle medium (DMEM) with high glucose-containing L-glutamine (Welgene, Gyeongsan-si, Gyeongsangbuk-do, Korea), supplemented 10% FBS, 100 U/mL penicillin, and 100 μg/mL streptomycin (all purchased from Welgene) [24]. 

### 4.2. Spinal Cord Injury Model and Transplantation Using the Intrathecal Catheter

All animal experiments were performed in accordance with the guidelines of the CHA University IACUC (Institutional Animal Care and Use Committee; IACUC090012). Adult male Sprague-Dawley rats weighing 270 g to 300 g were used in this experiment. Anesthetized rats by administration of ketamine (30 mg/kg, i.p) and xylazine hydrochloride (4 mg/kg, i.p) received laminectomy at the T11 level, followed by contusion with a 10 g rod dropped from 50 mm height using an NYU (New York University) impactor [58]. For cell transplantation, rats were implanted with catheters at 6 days before contusive SCI. One week after the injury, randomly selected SCI rats were transplanted with 1 × 10^6^ F3.BDNF cells in a total volume of 10 μL (n = 13) through sterilized PE-10 tubing (catalog no. 427401, BD Biosciences, Franklin Lakes, NJ, USA) that was threaded through an incision in the atlanto-occipital membrane to the T9 level. In the sham group, an equal volume of culture medium without cells (n = 8) was injected into the same site. The bladders were manually squeezed twice a day until spontaneous urinary extravasation. All rats received cyclosporine A (20 mg/kg) intraperitoneally for immunosuppression from 1 day prior to transplantation, and after transplantation, the dose was reduced to half and was maintained until sacrifice.

### 4.3. In Vivo MRI Detection of Transplanted Cells

All rats were examined to detect whether Feridex^®^-labeled F3.BDNF (n = 5) can migrate toward the injured region in the spinal cord using a 4.7 T BioSpec (Bruker, Germany) with T2- and T2 *-weighted imaging techniques. MRI examination was carried out following transplantation and afterward weekly for up to 10 weeks. T2-weighted images were applied to assess the full extent of the damaged spinal cord by contusion. T2 *-weighted images were also applied to understand the migration of stem cells following transplantation. Prior to cell transplantation and MR imaging, we confirmed the F3.BDNF cells labeled with Feridex^®^ by Prussian blue staining.

### 4.4. Immunocytochemical and Immunohistochemical Analyses

After 10 weeks post-transplantation, rats were transcardially perfused with heparinized saline (0.9% NaCl), followed by 3.7% formaldehyde. Subsequently, isolated brains and spinal cords were fixed overnight in 3.7% formaldehyde at room temperature and then transferred to 30% sucrose with shaking from 24 h to 72 h at 4 °C The brains and spinal cords were frozen in embedding medium (O.C.T. compound, Sakura Finetek, Torrance, CA, USA). Sections were incubated in blocking solution (5% normal horse serum (Vector Laboratories, Burlingame, CA, USA) in PBS containing 0.3% Triton X-100 (Sigma-Aldrich, St. Louis, MO, USA) for 1 h at room temperature. The following primary antibodies were used: Anti-human-specific nuclei (hNu) (mouse monoclonal 1:100, Millipore, Billerica, MA, USA), Anti-human-specific mitochondria (hMito) (rabbit polyclonal, 1:250, Millipore), Anti-NeuN (mouse monoclonal,1:500, Millipore), Anti-O4 (mouse monoclonal, 1:250, Millipore), Anti-GFAP (mouse monoclonal, 1:500, BD Biosciences), Anti-MBP (rabbit polyclonal, 1:250, Millipore), Anti-GABA (rabbit polyclonal, 1:5000, Sigma-Aldrich), Anti-human brain-derived neurotrophic factor (BDNF) (mouse monoclonal antibody, 1:200, R&D Systems, Minneapolis, MN, USA), ED1 (mouse monoclonal, 1:250, Serotec, Kidlington, UK), Iba-1(rabbit polyclonal, 1:250, Wako, Richmond, VA, USA), NOS2 (iNOS, rabbit polyclonal, 1:250, Santa Cruz Biotechnology, Dallas, TX, USA), CD206 (goat polyclonal, 1:100, Santa Cruz Biotechnology), CD86 (rabbit polyclonal, 1:200, Novus Biologicals, Centennial, CO, USA), FG (rabbit polyclonal, 1:5000, Millipore). After washing with PBS, the spinal cord sections were incubated with fluorescence-conjugated secondary antibodies, such as goat anti-mouse IgG conjugated Alexa-488 and 555, goat anti-rabbit IgG conjugated Alexa 488 and 555 (1:250, Molecular Probes, Eugene, OR, USA), goat anti-mouse IgM conjugated Alexa-555 (1:200, Molecular Probes) and Alexa Fluor^®^ 594 streptavidin (1:500, Jackson Immuno Research, West Grove, PA, USA) against the approximate species for 2 h. The sections were counterstained with the nuclear marker 4’, 6-diamidine-29-phenylindole dihydrochloride (DAPI). Afterward, fluorescence-labeled specimens were viewed and photographed under a confocal laser-scanning microscope (LSM510; Carl Zeiss Microimaging, Inc., Thornwood, NY, USA).

### 4.5. Prussian Blue Staining

Prussian blue staining was used for the detection of iron within the F3.BDNF cells labeled with Feridex^®^-protamine sulfate complex, which induced a reaction of ferric iron to the ferrous state with the formation of blue precipitates. F3.BDNF cells were incubated with Feridex^®^-protamine sulfate complex in the medium for 12–16 h at 37 °C. The medium was removed, and the cells were washed twice with PBS to remove any residual Feridex^®^-protamine sulfate complex. F3.BDNF cells were fixed for 15 min using 3.7% formaldehyde (Sigma-Aldrich). Cultures were then washed three times with PBS and incubated with Perls’ reagent (4% potassium ferrocyanide in distilled water, Sigma-Aldrich/20% HCl, Sigma-Aldrich, 50:50 *v*/*v*) for 30 min at RT. The tissues were then counterstained with eosin and observed using an optical microscope (Eclipse E600, Nikon, Tokyo, Japan). 

### 4.6. Histological Analyses

Transverse spinal cord sections were used to determine the extent of demyelination. Myelin sheath was visualized by luxol fast blue (LFB) staining, consisting of luxol fast blue 1.0 mg (Sigma-Aldrich), 95% alcohol 100 mL (Merck), 10% acetic acid 5.0 mL (Duksan, Seoul, Korea). Tissue sections were immersed for overnight at 60 °C. The sections were then washed with distilled water, followed by differentiation in 0.05% lithium carbonate (Sigma-Aldrich). Tissue sections were then counterstained with eosin and mounted. Serial longitudinal sections of the spinal cord were cut into 14 μm sections and examined by H&E staining. Volumes of transverse or longitudinal sectioned areas were measured using Image J (National Institutes of Health, Bethesda, MD, USA). The individual sub-volumes were obtained by multiplying the sectioned areas between sections, and the sub-volumes were summed to generate the total volume of cavities or spared white matter. 

### 4.7. Behavioral Tests and Electrophysiological Recordings

To evaluate the motor function of hindlimb, 2 methods were used. First, the locomotor function was assessed by using the Basso, Beattie, and Bresnahan (BBB) Locomotor Rating Scale [22]. Briefly, the BBB test was scored from 0, indicating no observable hindlimb movement, to 21, which exhibited a consistent and coordinated gait with a parallel paw placement of the hindlimbs and consistent trunk stability. Second, the degree of motor deficit was estimated by the combined behavioral score (CBS) [23]. Tests were conducted on the following items: motor scores, toe spread, righting reflex, extension withdrawal reflex, placing reflex, and inclined plane, totally scored from 0 for a normal rat to 90 for a completely paralyzed rat. Pain-related behavior as a test for the level of neuropathic pain was recorded to evaluate the vocalization threshold to graded mechanical touch/pressure using the von Frey filaments, ranging from 10 g to 300 g (i.e., 10, 15, 26, 60, 100, 180, 300 g; Stoelting, Wood Dale, IL, USA) [25,59]. The intensity of the stimuli that induced consistent vocalization was considered as the pain threshold and applied 5 times during the test from each rat [59]. The motor-evoked potentials (MEPs) were examined to measure electrophysiological conduction at 10 weeks after transplantation of F3.BDNF cells in SCI. All rats were anesthetized and tightly fixed to a stereotaxic apparatus. Scalping and craniectomy measuring 4 × 4 mm were performed with a drill in the right frontoparietal area. Silver ball electrode was placed epidurally in the right frontoparietal area via the holes. The MEP was elicited by stimulating the right temporalis muscle with a square pulse (0.1 ms, 5 mA, 3 Hz) which was made by a signal isolator (A365, WPI, FL, USA) and pulse generator (Pulsemaster A300, WPI, Sarasota, FL, USA). It was recorded from the left gastrocnemius muscle, and amplified (gain: 10,000, bandpass: 300–1000 Hz) by a bioamplifier (ISO-80, WPI) and transducer amplifier (TBM4M, WPI). The amplified waves were delivered to a computer by an AD converter (1401, CED, Cambridge, UK), and averaged 60 epochs by software (Spike 2, CED). Recording needle electrode was placed in the tibialis anterior muscle.

### 4.8. Anterograde and Retrograde Tracing

At 10 weeks after transplantation of cells, we performed anterograde and retrograde tracings to analyze the formation of contacts between the host neuron and transplanted F3.BDNF cells. For anterograde labeling, 1 μL of a 10% solution of biotinylated dextran amine (BDA 10,000, Molecular Probes, USA) were injected into the location of the hindlimb motor cortex (coordinates: 2.0 mm posterior to bregma, 2.0 mm lateral to bregma, 1.5 mm depth) using a glass micropipette [60]. BDA injection was performed at a constant flow rate of 0.1 μL /min to diffuse to the cortical area and the animals were remained in place for 2 min after the completion of injection. At 2 weeks after BDA injection, the transplanted group (n = 3) was perfused with 3.7% formaldehyde. For the injection of retrograde tracers, rats were injected with a 5% solution of Fluoro-Gold (FG) (Molecular Probes) retrograde tracer using a glass micropipette. 0.5 uL of FG was injected bilaterally at a point 0.5 mm lateral to the posterior-median vein and a depth of 1.2 mm and the bilateral dorsal root entry zones [60,61]. Subsequently, rats underwent laminectomy at L1 and complete spinal cord transection using micro-scissors [62]. At 2 weeks after BDA injection and 1 week after FG injection, F3.BDNF cell transplanted group (n = 3) was sacrificed and fixed with 3.7% formaldehyde for immunohistochemical examination.

### 4.9. Statistical Analysis

Statistical analyses were performed using SPSS (v 15). All values were presented as mean ± S.E.M (standard error of the mean). The Friedman test was used to compare scores for the BBB, CBS, and nociception test scores obtained on a given experimental day between 2 groups (Sham group vs. F3.BDNF group). Differences in marker expression were calculated by a Student’s *t*-test. The probability value less than 0.05 were considered to be significant.

## Data Availability

The data that support the findings of this study are available from the corresponding authors upon reasonable request.

## Supplementary Materials

The following are available online at https://www.mdpi.com/article/10.3390/ijms22136970/s1.
