# Supplementary material for: Therapeutic Effect of BDNF-Overexpressing Human Neural Stem Cells (F3.BDNF) in a Contusion Model of Spinal Cord Injury in Rats
Source: Int J Mol Sci. 2021 Jun 28;22(13):6970. doi: 10.3390/ijms22136970 (PMC8269438; doi:10.3390/ijms22136970)
Supplement: Supplementary file 1 [file ijms-22-06970-s001.zip › ijms-1216552-supplementary.pdf]

Supplementary Materials

Figure S1. Prussian blue staining showing the detection of F3.BDNF cells labeled with Feridex®)

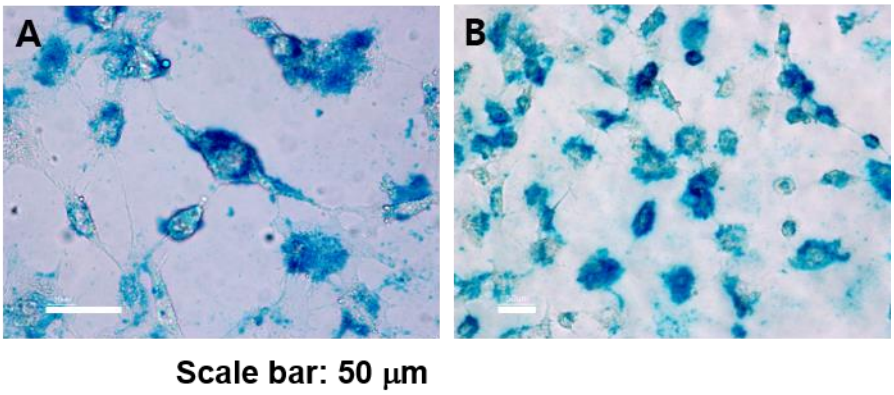

(A) High magnification (10x), (B) Low magnification (4x). The blue staining in the cytoplasm showed the presence of iron particles. Scale bar: 50 μm

Figure S2. Changes of GFAP expression by transplanted F3.BDNF cells

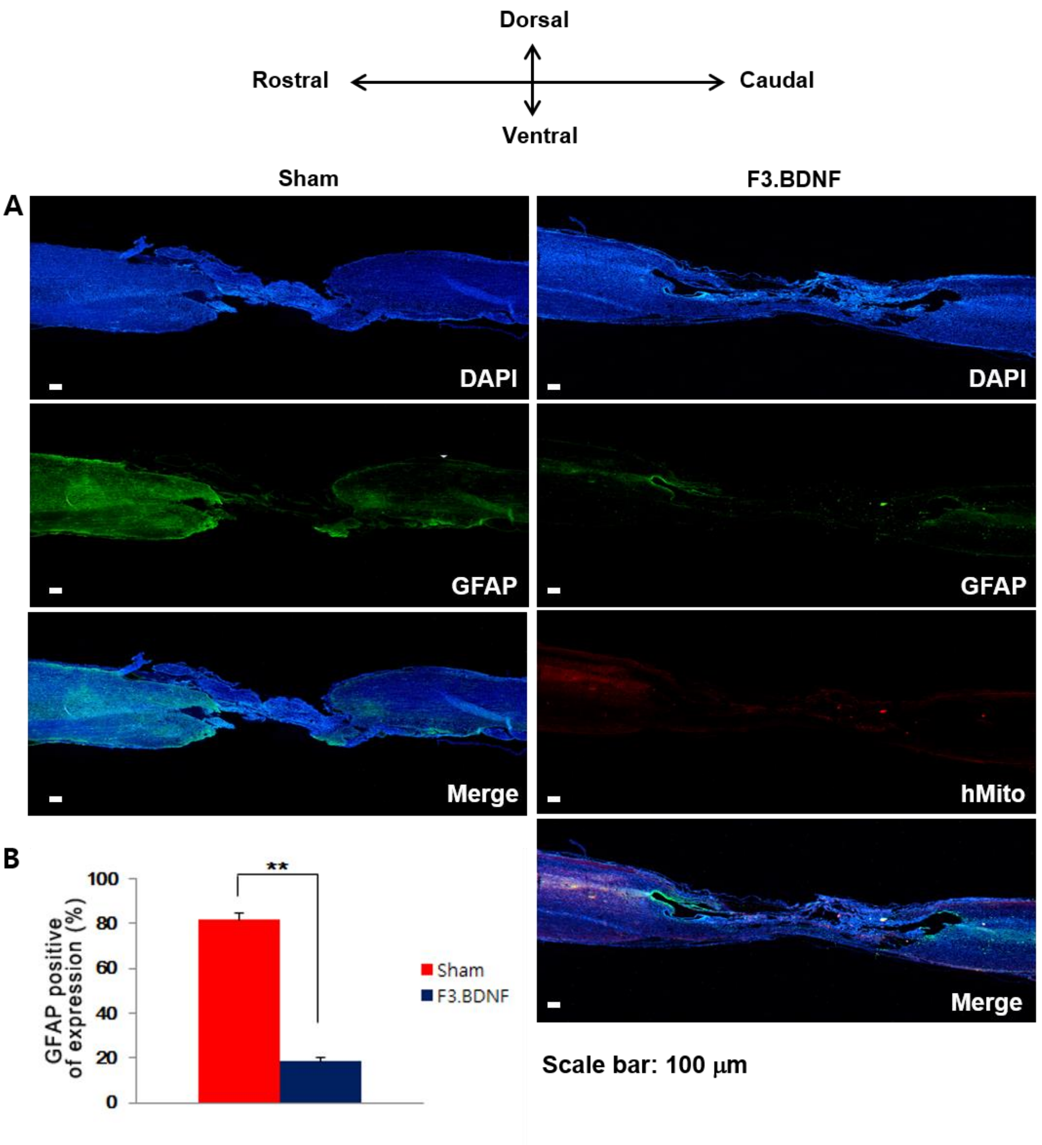

(A) GFAP-positive cells (green) were detected in the parenchyma of injured spinal cord (left panels), which became decreased after cell transplantation (right panels). Small numbers of transplanted human cells were also observed by immunofluorescence staining against hMito (red). (B) Quantitative analysis showing a significant decrease in the number of GFAP-positive astrocytes after F3.BDNF cell transplantation in spinal cord injury. *P < 0.05, Scale bar: 100 μm

Figure S3. Immunohistochemical staining showing co-localization of hNu- (red) and GABA- (green) positive cells in the injured spinal cord

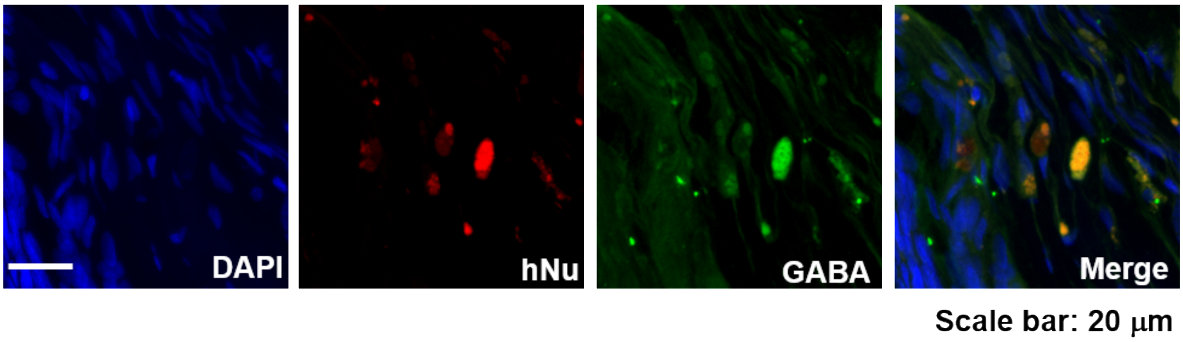

Scale bar: 20µm. Abbreviations: GABA, gamma-aminobutyric acid

Figure S4. Increased GABA expression below the level of contusive spinal cord injury following F3.BDNF cells transplantation

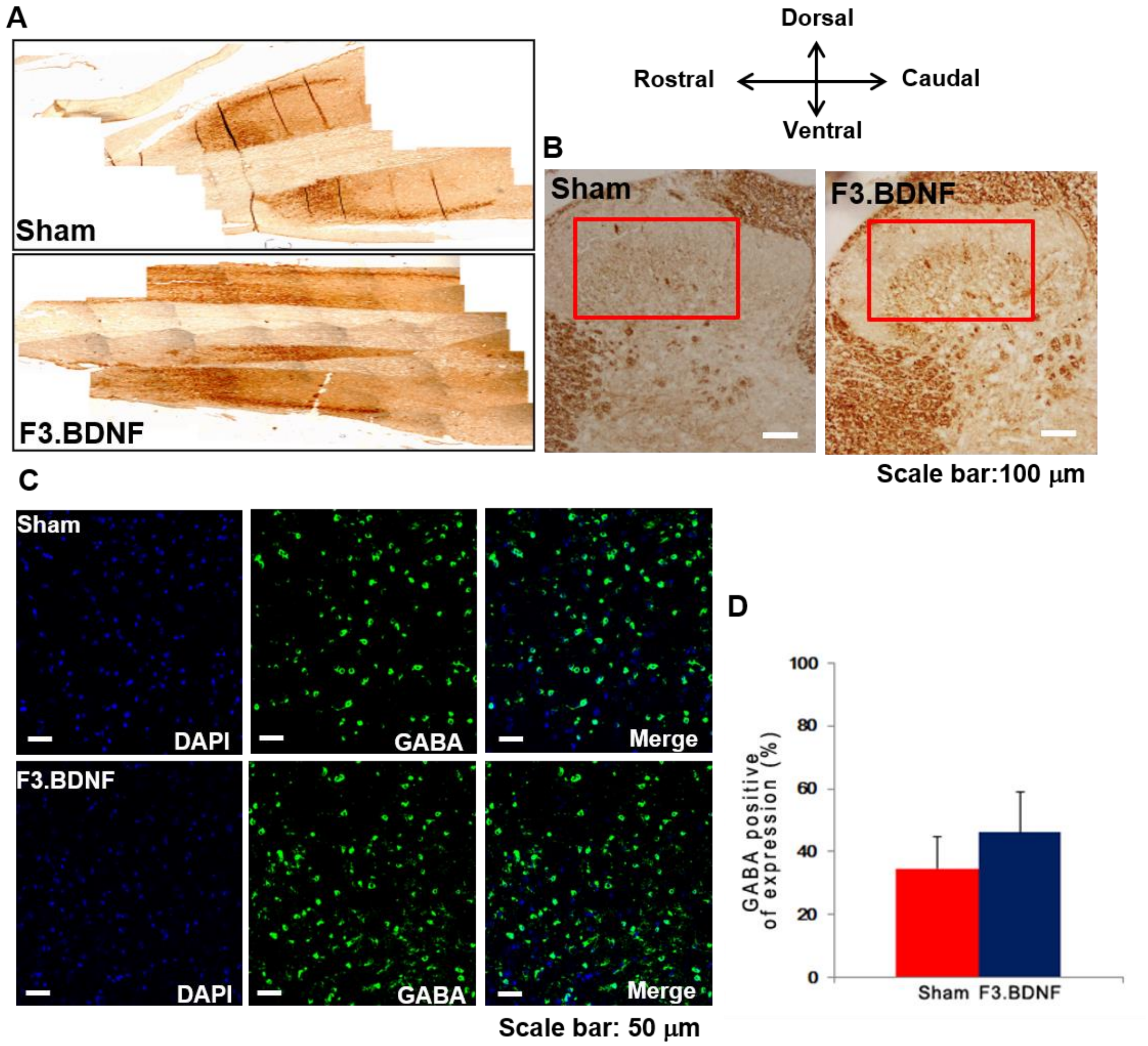

(A) Low magnification showing GABA expression on horizontal section. Expression of GABA, an inhibitory neurotransmitter, was induced in the transplanted F3.BDNF group compared to the sham group. (B) DAB staining showing a difference of GABA expression in the lamina II of spinal dorsal horn at T13. (C, D) Immunohistochemical staining and its quantification showing no significant difference in GABA-positive cells between two groups in spinal dorsal horn. Scale bars: 50µm. Abbreviations: DAB, 3, 3' Diaminobenzidine.
